# Potential Protein Production from Lignocellulosic Materials Using Edible Mushroom Forming Fungi

**DOI:** 10.1021/acs.jafc.2c08828

**Published:** 2023-03-08

**Authors:** Karin Scholtmeijer, Lambertus A. M. van den Broek, Arnout R. H. Fischer, Arend van Peer

**Affiliations:** † Wageningen Plant Breeding Research, Mushroom Research Group, Droevensdaalsesteeg 1, 6708PB Wageningen, The Netherlands; ‡ Wageningen Food and Biobased Research, Bornse Weilanden 9, 6708WG Wageningen, The Netherlands; § Wageningen University Marketing and Consumer Behaviour Group, Hollandseweg 1, 6706KN Wageningen, The Netherlands

**Keywords:** mushroom, substrate mycelium, lignocellulose conversion, protein transition, food products, circular economy, biobased economy

## Abstract

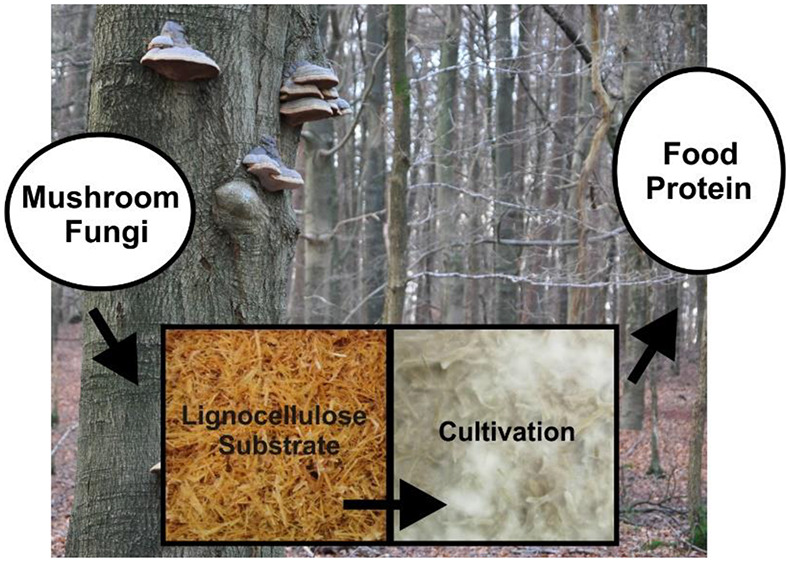

There is a need for new protein sources to feed the world in a sustainable way. Converting non-food-grade “woody” side streams into food containing proteins will contribute to this mission. Mushroom forming fungi are unique in their capability to convert lignocellulosic substances into edible biomass containing protein. Especially if substrate mycelium can be used instead of mushrooms, this technology could be a serious contribution to addressing the protein challenge. In this Perspective, we discuss challenges toward production, purification, and market introduction of mushroom mycelium based foods.

## 1 Introduction

Protein is a key ingredient in human nutrition. To feed a global population that is expected to have grown to 10 billion by 2050, there is an urgent need for additional protein sources. Current (animal) protein production systems are often not sustainable and continuing expansion is not a viable option. To achieve a climate neutral world, present and future protein production are hence one of the issues to be brought in line with (among others) the Paris Agreement^1^ and the UN Sustainable Development Goals.^2^ In other words, new and additional sources for protein are required.

Different approaches are needed to meet sustainability goals and create more protein, such as increasing agricultural production with less pressure on the environment, reducing food spoilage, more efficient use of biomass resources, and diversion from animal protein-based diets (meat and dairy) toward more sustainable protein sources. Especially in high-income countries,^3^ consumer interest in non-animal-based proteins has been newly increasing, and novel sources of protein such as insects, cultured meat, legumes and other plants, microalgae, seaweed, and mycoprotein (often total fungal biomass) are explored.

Until recently, mycelium-based food products were few and most notably concern imperfect fungi grown on nutrient rich liquid broths. For example, Quorn is produced from the ascomycetous fungus *Fusarium venenatum*(4) grown on food grade, starch rich liquid, resulting in a product that contains ∼12% (w/w) protein and ∼5% (w/w) fiber. To improve sustainability and circularity of protein production, employing non-food-grade, underutilized, and preferably renewable resources would be optimal. Lignocellulosic side streams from agriculture, forestry, and industrial processes are as such very appealing, offering a wide range of materials that have little application yet, like cocoa husk, nutshells, peels, press cakes, cuttings, wood chips, and so on. Use of such side streams for food or feed is limited because most organisms, including most imperfect fungi, cannot cope with lignin, a very recalcitrant component of lignocellulose.

However, one group of fungal species, the basidiomycetes, is capable of efficiently converting lignin rich side streams into fungal biomass that contains protein. Basidiomycetes include most of the mushroom forming fungi, and many grow on wood or leaf litter, i.e., lignocellulosic substrates. Technically, basidiomycetes could thus be used to generate protein from lignocellulose side streams. An additional advantage is that such fungal cultivation does not have to compete with arable land (needed for nature and animal and plant protein), as fungal cultivation can be performed indoors.

About 2000 mushroom species are reported to be edible, of which a number have a GRAS (Generally Regarded As Safe) status. However, the majority of mushroom species has never been considered for consumption because of small size, off taste, or unpleasant texture or odor of the mushrooms. Nevertheless they are nontoxic and could be consumed. Mushrooms are considered to be healthy since they are low in calories, sodium, and saturated fats while they are high in fiber and contain vitamins and trace elements. Beyond nutritional qualities, also health promoting properties of mushrooms have been indicated.^5^ Unfortunately, commercial scale production of mushrooms is challenging and has only been established for about 80 species. Producing protein with substrate mycelium of mushroom fungi, instead of producing mushrooms for protein would encompass several advantages. (I) Substrate mycelium of many mushroom forming fungi can be conveniently cultured on lignocellulose while available methods for producing their fruiting bodies are very limited. (II) Without using fruiting bodies, production cycles will be shorter, and less energy intensive culturing conditions and facilities (no special climate for fruiting) are needed. (III) Fungal species could be selected based on their protein content and growth or conversion efficiency without the limiting requirement of existing or newly developed mushroom production systems. Finally, (IV) large amounts of substrate mycelium remaining after mushroom cultivation can serve as a protein source for food instead of or before its use for other applications.

Despite the possible advantages, the substrate mycelium has until now largely been neglected as an alternative source for protein that can be generated on lignocellulosic side streams. Also, it would be classified as a novel food, and its nutritional value is largely unknown. Literature presents confusing data on fungal protein content, properties, and nutritional values, due to among others use of (often incorrect) nitrogen–protein correction factors and comparison of uncontrolled samples. Unfamiliarity with methods for production, harvesting, and processing of mycelium and protein of mushroom fungi prevent benefit assessment and marketing potential, hampering investments to develop this resource. There have been very few risk assessments for approval of mycelia for food.

**Figure 1:**
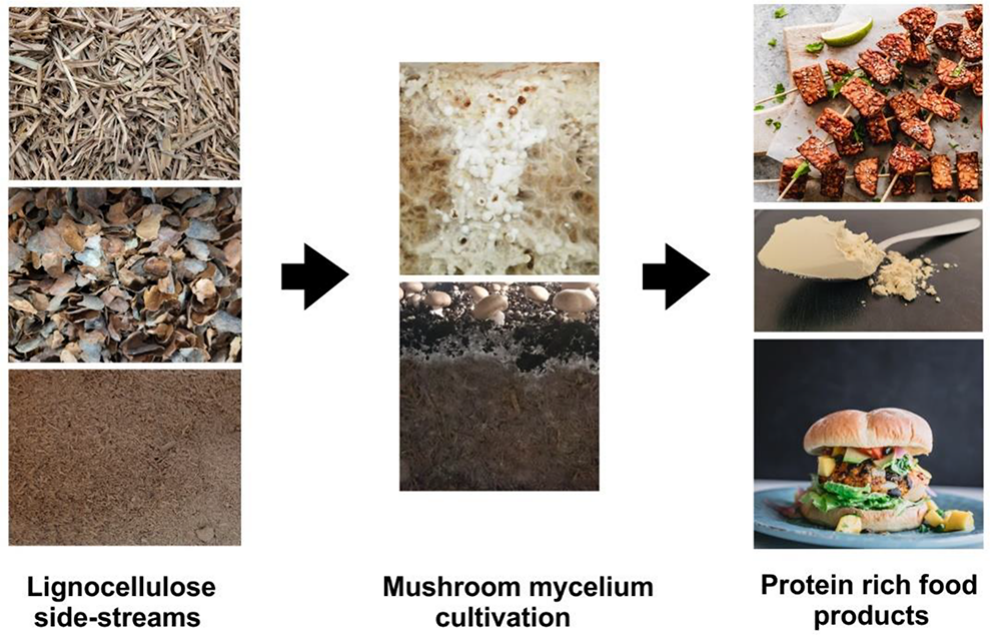
Conversion of lignocellulosic side streams into protein rich food products using cultivation of mycelium from mushroom forming fungi. Please note that the picture of tempeh merely serves as an example for similar products based on other substrates, which may not yet be food grade. Parts of the figure are used with permission from Unsplash: https://unsplash.com/license (Image of tempeh by Ella Olsson, 2019. Image veggie burger by Deryn Macey, 2018).

To shed light on the scattered and contradictory information, this Perspective aims to take a position and prioritize the most relevant possibilities and challenges of lignocellulosic substrates as a sustainable source for protein via the cultivation of substrate mycelium from mushroom fungi (Figure 1). We will discuss (I) what is known about proteins from mushroom forming fungi, (II) important aspects of cultivation of mushroom fungi, (III) harvesting and processing of substrate mycelium of mushroom fungi from lignocellulose, and (IV) to what extent and under what conditions consumers may respond favorably to the use of these mycelia grown on lignocellulosic materials for food.

## 2 Protein from Mushroom Forming Fungi

**Figure 2:**
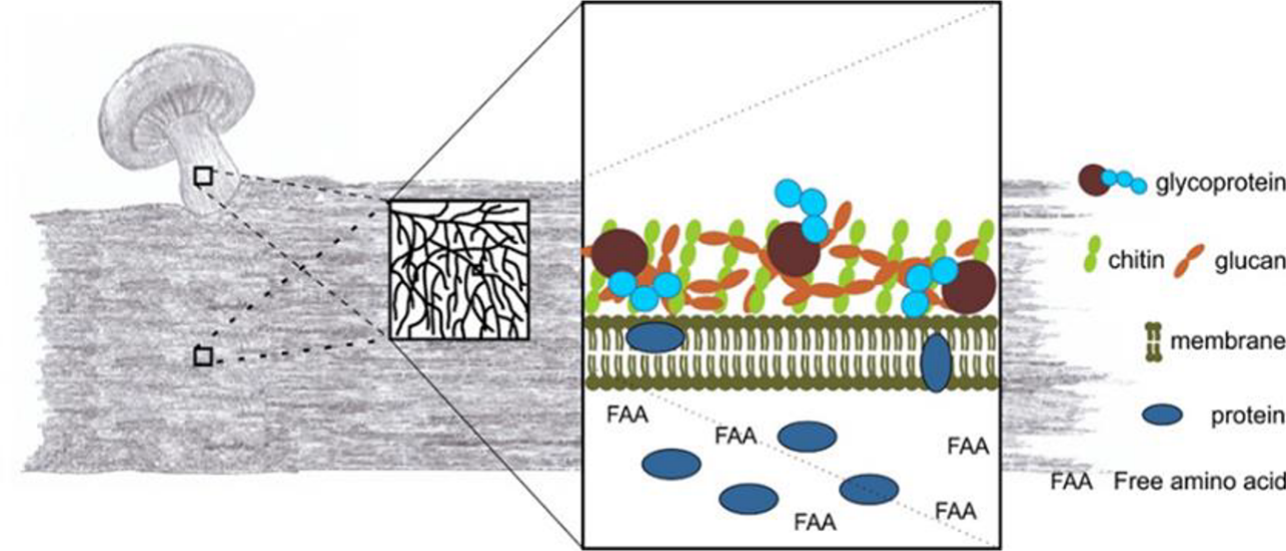
Schematic overview of mycelium, either growing in a substrate or making up a mushroom. All structures consist of a network of hyphae, but individual hyphae do differentiate according to their specific functions. All hyphae contain protein, free amino acids, and other nitrogen containing compounds such as chitin, nucleic acids, urea, ammonia, amines, quaternary ammonium compounds, volatile nitrogen compounds, and vitamins. Proteins are bound to the cell membrane or the fungal cell wall, are present in the cytoplasm, or are secreted into the substrate.

Literature on the content, quality, and nutritional value of protein from mushroom forming fungi almost exclusively considers their fruiting bodies, i.e., mushrooms, as these are typically consumed while the remaining spent substrate containing mycelium is discarded. Although substrate mycelium of mushroom fungi contains protein as well (Figure 2), only recently the consumption of substrate mycelium of mushroom fungi is being explored.

Since the bulk of literature is focused on composition of mushrooms, we discuss what is known for these proteins. Mushrooms are often described as being high in protein,^6,7^ yet reported estimates on protein levels range from 4% up to 57% in dry weight (DW). Traditionally, protein contents of mushrooms are calculated based on their mineral nitrogen content, multiplied by a N-to-protein conversion factor of 6.25. This procedure will be the same for mycelium and for fruiting bodies. The conversion factor has been reduced to compensate for existing non-protein nitrogen compounds to 4.38^8^ but was certainly not incorporated in all following research. Mushroom forming fungi contain considerable amounts of nonprotein nitrogen compounds, including chitin, nucleic acids, urea, and ammonia. Moreover, amines, quaternary ammonium compounds (ammonium salts), volatile nitrogen compounds, and nitrogen-containing vitamins can be found in fungi.^9,10^ All these nitrogen sources are included using Kjeldahl based protein analysis, but their levels and proportions can vary largely between fungal species or strains of the same species, as well as depend on cultivation conditions or the developmental stage of the mushroom or the mycelium.^7^ Present reports on protein contents of fungal species often are based on a single strain, while the analyzed fruiting bodies originate from a wide variety of unidentified substrates or simply “from the wild”. Therefore, applying a general N-to-protein factor based on total nitrogen (Kjeldahl) can quickly result in inaccurate protein estimates,^10^ and care should be taken when making comparisons of separately reported protein contents of different fungal species.

More accurate protein content determination for mushrooms and/or mycelium involves analysis of the amino acids (AA). After measuring total amino acids (TAA), free amino acids (FAA) that are not part of proteins should be determined and subtracted from the TAA. Furthermore, the existence of peptide bonds in proteins should not be overlooked during calculations of protein amounts.^11^ Distinction between FAA and TAA is especially important when considering fungal protein for technofunctional and/or functional properties and for accurate determination of nutritional values, as digestibility, for example, will be different. Here also, care should be taken which samples are analyzed and compared as it is largely unknown how and at what efficiency mushroom fungi produce protein in relation to their specific substrate and which other parameters influence this process.

In summary, there is general agreement that mushrooms and substrate mycelium from mushroom fungi contain protein and that this can be determined by AA analyses but that actual protein and AA contents and compositions are not well established for both substrate mycelium and fruiting bodies of these fungi. Both do contain protein, free amino acids, and other nitrogen containing compounds, but they may be expected to differ in amount and composition since they represent different structures or developmental stages. Proteins can be bound to the cell membrane or the fungal cell wall, are present in the cytoplasm, or are secreted into the substrate.

### 2.1 Protein Types and Protein Properties from Mushroom Fungi

It should be realized that “protein” from mushroom fungi always consists of a complex mixture of many different proteins. This is in contrast to plants or animals where certain parts can contain one or a few predominant (classes of) proteins like albumins, globulins, glutelins, and prolamines. Fungal proteins are known to be differentially expressed in tissues, growth stages, or developmental processes or in response to environmental factors (e.g., laccases, hydrophobins, and lectins), meaning that selecting different parts of a fungus will result in different protein mixtures.

While specific bioactivities for some proteins of mushroom fungi have been reported including lignocellulolytic enzymes, lectins, fungal immunomodulatory proteins (FIPs), proteolytic enzymes, protease inhibitors, ribosome inactivating proteins (RIPs), ribonucleases, oxidoreductases, biotin binding proteins, membrane-pore-forming proteins, and various antimicrobial (bacteria, fungi, viruses) proteins and peptides,^12^ their relative contribution to “total protein” is unknown.

Technofunctional properties of proteins from mushroom fungi for food applications are even less explored. Properties important for food such as oil absorption, emulsion, foaming, gelling and changing bulk density are currently only known from concentrates or powders obtained from whole mushrooms. Surely, the properties of these protein mixtures will vary between mushroom species, strains, and cultivation conditions and depend on preparation methods.^6,13^ Thus, far, only one specific class of proteins produced by mushroom fungi, the hydrophobins, has been examined in detail for technofunctional properties. They are unique to fungi, small, and relatively hydrophobic. All known hydrophobins are capable of self-assembly on hydrophobic–hydrophilic interfaces and can be used to stabilize foams and emulsions for food applications. In addition, different hydrophobins vary in solubility, foam stability, and emulsion capacity.^14^ Undoubtedly, mushroom fungi carry a far larger potential for technofunctional and biologically active proteins that have yet to be discovered. Similarly, many beneficial health aspects (e.g., immunomodulatory proteins), effects on nutritional value (proteolytic enzymes), or (serious) detrimental effects (toxins) of fungal proteins remain to be determined.

Overall, mushroom fungi do contain a complex assortment of proteins with potentially very interesting functions and applications that could be of interest in pure form. With respect to total protein, properties will need to be carefully analyzed in relation to specific growth and developmental conditions of the fungus.

### 2.2 Nutritional Value of Protein in Mushroom Fungi

Differences in nutritional values of protein from various mushroom species are reported^6^ as ranging from poor to very high.^15^ Methods estimating nutritional values of mushroom proteins vary from calculations based on crude protein levels (Kjeldahl based), amino acid (AA) composition profiles (mostly total amino acids; TAA), essential AA (EAA), standardized *in vitro* protein digestibility (IVPD), protein digestibility-corrected amino acid scores (PDCAAS), and combinations of these methods complemented with dietary experiments in animals.^6,15^ Determination of actual protein levels is, of course, crucial when studying nutritional values but is rarely done. FAA are generally more easily taken up by the digestive system than AA as part of proteins, especially if such proteins exhibit resilience toward enzymatic degradation. Meanwhile, FAA can make up as much as 50% of the amount of TAA^9,10^ in mushroom fungi. This is higher in comparison to most other protein crops^16^ and could be considered beneficial for uptake efficiency. Glutamic acid and α-alanine are most abundant, but also significant amounts of proline, glutamine, arginine, and aspartic acid have been detected.^9^ One approach to obtain relevant estimates for nutritional values of the AA can be comparing AA-profiles and amounts to WHO/FAO recommendations for maintaining health (2011).^8^ It is interesting to note that Ayaz^17^ reported the TAA profiles of 11 edible mushroom species, each containing sufficient levels of EAA per gram of protein according to WHO/FAO recommendations (the percentage of EAA is compared to the percentage of the EAA in a control protein given by WHO/FAO).

The nutritional value of actual proteins in mushroom fungi will be, to a large extent, influenced by the availability and digestibility of the different proteins, and thus by the presence of antinutritional compounds.

Finally, processing of mushroom fungi prior to consumption via heating, canning, extraction, or purification will affect the nutritional value and change amino acid profiles and protein conformations.^18^ Estimates on nutritional values of fungal protein should therefore be best applied on the products that are consumed and not just on the protein content of the substrate mycelium or fruiting body.

Overall, AA compositions of mushroom fungi contain all EAA in sufficient amount,^6^ and a considerable part of the AA is in free form which might be beneficial from a nutritional perspective. Regarding the nutritional value of the proteins, little is known, and effects of special bioactive proteins within the mixtures of total protein will need attention, as will contents of antinutritional compounds.

## 3 Cultivation of Mushroom Fungi on Lignocellulosic Substrates

In solid state systems, mushroom-forming fungi colonize substrates through hyphae, forming a network called mycelium (mycelium is generally used for the hyphae present in the substrate, while mycelia making up fruiting bodies are dubbed mushroom) which degrades the substrate and takes up released nutrients. Under the right environmental conditions, selective parts of the mycelium can aggregate and assemble into (for many mushroom species) macroscopic structures on the surface, i.e., mushrooms (Figure 2). In general, for commercial mushroom cultivation, a lignocellulosic substrate (e.g., compost, straw, or sawdust) is pasteurized or sterilized, supplemented with additional nutrients, and inoculated with spawn (liquid or grains) containing the desired fungus. During colonization, the fungus will partially degrade the substrate using a battery of lignocellulolytic enzymes,^19^ thereby eliminating part of the dry matter through respiration and converting another part of the dry matter into fungal biomass including chitin, glucans, and protein. After colonization of the substrate, fruiting bodies are induced by precise and specific climate changes in the cultivation setting. These specific climate conditions often involve cooling and ventilation and can be energy intensive. At this moment, the fungus will change its metabolism and extract more nutrients from the substrate to produce the mushrooms. Thereafter, the mushrooms are harvested (1 or multiple flushes), and the remaining colonized substrate is discarded as a side stream.

Lignocellulose consists of 5 main components, cellulose (35–50%), hemicellulose (20–35%), lignin (10–25%), ash, and protein (up to 10%) on dry weight basis.^19^ Especially lignin is often a limiting factor in utilization of lignocellulose for many organisms. Most mushroom forming fungi can open the lignocellulosic matrix and thereby access the more nutritious cellulose and hemicellulose. It should, however, be realized that the extent to which lignocellulose serves as a source of nutrients for the growing fungus is highly dependent on the mushroom cultivation system and the substrate that is provided. Mushroom factory systems (bottle cultures) for exotic mushrooms are characterized by short production cycles and single harvests and depend on heavily nutrient enriched sawdust. In such systems, the fungi predominantly grow on the added nutrients, while the sawdust is only degraded to a very low extent and mainly serves as a water holding matrix. In other cultivation systems using straw-based substrates and composts the fungus will consume a considerable part of the lignocellulose but is helped by some added nutrients to optimize yields. These systems usually comprise two or three harvests. Finally, in wood-log cultivation systems, all of the nutrients are derived from the lignocellulose by the fungus. Wood-log cultivation is characterized by long production cycles (1 cycle a year) over multiple years. The same dynamics should be considered when developing protein production systems with mycelium of mushroom fungi based on conversion of lignocellulose, with faster growth usually requiring higher levels of added nutrients.

While mushroom forming fungi are technically capable of growing on lignocellulosic substrates in liquid culture systems, research has mainly focused on sugar rich side streams which also contain lignocellulose, like apple pomace or beet molasses.^11^ In such systems, little of the actual lignocellulose is utilized by the fungus, mimicking the highly supplemented solid-state fermentation for mushroom production in bottles, and the production of fungal biomass in nutrient broths like Quorn. Liquid culture systems in which lignocellulose is the actual source of nutrients for production of fungal biomass are unknown to the authors.

Considering lignocellulosic side streams as a resource for cultivation of mycelium of mushroom fungi, a rough calculation indicates an enormous potential for expansion. For 2013, it was estimated that 5 billion tonnes of agricultural residues were produced worldwide. The largest part consisted of lignocellulosic residues from cereals, oil crops, and sugar cane.^20^ Meanwhile, up to 9 million tonnes^21^ of mushrooms are produced annually worldwide. Assuming between 10 and 30% bioefficiency in mushroom production (ignoring supplemented nutrients), it would require 90 to 270 million tonnes of lignocellulosic residue to sustain the world mushroom production. This is less than 6% of the total available agricultural residues. Moreover, the estimated agricultural residues did not include wood cuttings and sawdust. These can also be (and already are) used for cultivation of mushrooms and mycelium, increasing the total amount of lignocellulosic residues available for generating protein with fungi even further.

Current lack of knowledge on how to efficiently produce the fruiting bodies of many (edible) species is limiting the use of many side streams for growing fungi. Utilizing mycelium without producing mushrooms is far more feasible, enabling selection from a much wider range of mushroom species for protein production adapted to specific lignocellulosic side streams. Combined with shorter production cycles and less complicated environmental regimes, this could increase the efficiency of using agricultural, forestry and industrial side streams (in number, amounts, time, and location) to produce fungal protein. Cultivation technology and infrastructure for growing mushroom fungi in bulk on lignocellulose already exists, e.g., the tunnel systems that are used in preparation of colonized composts for the Button mushroom (or the bottle systems employed mainly in Asia).

To allow estimations on economic feasibility of protein production using mycelium, Button mushroom substrate production may serve as an example. Fully grown substrate has a bulk density of 450 kg·m^3^ and about 6.8% of this is mycelium material.^22^ Thus, 1 m^3^ of grown substrate contains about 30 kg of mycelium. At a protein content of 2.3% of the wet weight^23^ (assuming a similar protein level in mycelium compared to mushroom), this would equal 690 g of protein which equals 1.5 kg/tonne of substrate. A typical cultivation tunnel of 125 tonne can thus deliver 187 kg of protein, assuming that all protein can be recovered. One tonne of mushroom colonized substrate is sold at €150 per tonne and thus one tunnel has a value of €18,750 resulting in the price of the unprocessed protein of €125 per kg. For comparison, chicken filet costs €10 per kg and contains 23.3% protein resulting in €43 per kg chicken protein. Additional processing needed to extract the protein from the substrate will further add to the price per kg. Still, without any optimization for protein thus far, using fungi on lignocellulose to produce protein is not so far from prices for protein by highly optimized systems as chicken protein. This suggests that improvements in substrate–mycelium combinations resulting in higher bulk densities or otherwise increased amounts of protein per kg of substrate will lower the price to levels that can compete with animal protein.

As stated, the amount of fungal protein that can be produced on lignocellulose and in what time span remains to be more accurately determined and awaits improvement and optimization. Effects of even simple technical improvements like adding more nitrogen to the substrate on the protein content of the fungus are largely unknown. However, they offer an interesting scenario for combining nutrient-rich side streams (especially those with excess of nitrogen) with lignocellulose to optimize fungal protein production. In conclusion, lignocellulose side streams offer a vast and renewable resource to produce mycoproteins,^11,15^ economic feasibility of price per kg protein is not discouraging, and optimization of protein levels in the production with fungi might well bring such protein in reach as a true alternative.

## 4 Harvesting and Processing of Mushroom Mycelium

Fungal mycelium in general is mainly cultivated in liquid cultures (fermenters) on nutrient rich substrates. For more sustainable production, mushroom mycelium may be cultivated in liquid cultures provided that lignocellulosic substrates can be pretreated to obtain small size particles (blending, milling) in an economically viable and energy friendly manner. For solid state cultivation of mushroom fungi, knowledge and infrastructure are already in place (composting companies) which may be an advantage. For solid state fermented materials, different approaches are possible for using the resulting edible mushroom mycelium in food applications, such as using (I) a whole product, containing both substrate and mycelium, (II) the mycelium separated from the substrate, (III) the protein extracted from the whole product or from the mycelium isolated from the substrate, or (IV) only excreted proteins after isolation. When non-food-grade or otherwise not ready-to-eat substrates are used (e.g., wood chips, cocoa husks, press cakes), there are more limitations to use the mycelium as a protein source for food and processing will be of increasing importance.

(I) If food-grade substrates are used for the cultivation of edible mushroom mycelium, the substrate and mycelium could be used together as a food product, such as tempeh, to access the fungal protein. Products consisting of mycelium and edible lignocellulosic material can only be used as a novel food (Regulation (EU) 2015/2283). Here, it is important that the mycelium and the lignocellulosic substrate either have a food grade status or acquire this status through the process. Using such whole fermentation food products, it is also important that the texture and flavor of the final product is attractive. The food product can be used or consumed directly after sterilization or pasteurization. Lignocellulosic side streams can contain components that should only be present at low concentrations to have no negative health effects. For example, theobromine present in cocoa husks or pods is safe for humans at normal intake doses of cocoa, but is toxic for several (companion) animals.^24^ Presence of such compounds in whole fermentation food products or concentration of such compounds in the fungal protein after processing need careful consideration when selecting lignocellulosic substrates.
(II) Separation of the mycelium from the substrate will be more challenging and is dependent on how the fungus is attached to its substrate. It can be possible to cut slices of mycelium covering substrate when there is a clear transition state. An example of this method is the mushroom mycelium-based bacon alternative of MyForest Foods. However, this does not much differ from producing and harvesting mushrooms, and spent substrate with mycelium is still removed as a side stream. For utilizing substrate mixed with mycelium, separating mycelium from the substrate mycelium matrix will require rigorous methods or may even be impossible. No clear methods for separating mycelium grown in lignocellulose matrices are known.
(III) Proteins can be extracted from whole products (substrate with mycelium) or from the isolated mycelium. In the case of whole products, it will be more challenging to separate the proteins originating from mycelium and the substrate, if needed. For efficient protein extraction, cell disruption is especially important to release intracellular protein. This can be achieved by mechanical methods, such as grinding, high pressure homogenization, and (bead) milling, or more novel extraction technologies, like microwave-assisted, ultrasound-assisted, and pulsed electric field extraction (PEF).^25−27^ Non-mechanical/physical options include enzymatic treatment and chemical extraction with solvent, acid, and alkali. Conditions can be chosen that favor the release of proteins from mycelium instead of the substrate. If desired or needed, e.g., separation of the proteins of different origins, further fractionation steps such as filtration and pH treatment can be applied, and the final protein fraction can be stabilized by spray- or freeze-drying. Few of those methods have been actually tested for larger scale mycelium–substrate protein extraction. For further development, conditions and methods should be well chosen to avoid protein denaturation or adverse effects on nutritional values and AA profiles.
(IV) While growing, mushroom mycelium secretes proteins, often in the form of enzymes but also as low molecular mass proteins. These enzymes and proteins can be isolated by centrifugation or pressing of the mycelium–substrate matrix. The presence of lignocellulosic material in this case can be advantageous during pressing, functioning as a pressing aid, since the mycelium itself is ductile and this property is influenced by the substrate used.^28^

Depending on the approach used, protein fractions with varying protein composition will be obtained. These differences will also be reflected in the other components that may be present in the extracts, bringing the potential benefit of additional healthy fungal components or conversely carrying the risk of unwanted harmful compounds.

Material left over after processing of the mycelium–substrate matrix contains cell walls from mycelium and lignocellulosic substrate that, while not protein, could be interesting for valorization and help to make the protein production system viable. β-Glucans and chitin are the main polysaccharides present in the cell walls of mycelium while the substrates will also contain modified lignocellulose. For further valorization, fractionated β-glucans can be used for antimicrobial and anticancer applications, and chitin can be converted into (vegan) chitosan for medical and pharmaceutical use, packaging, agriculture, textiles, cosmetics, and water treatment.^29^ Modified lignin could find application as carbon fiber, composite material, antioxidant, UV absorbent agent, antimicrobial agent, or fire-retardant or for 3D printing.

Taken together, protein obtained from mycelium of mushroom forming fungi will consist of a broad mixture of different types of protein complemented by additional components (unless it is highly purified). Several routes seem possible depending on the exact substrate, although separation of whole mycelium from within substrate matrices is very challenging. To improve sustainability, circularity, as well as the economic viability of protein production through fungi, a biorefinery approach will be required for total valorization of all components used during fermentation.

## 5 Consumer Acceptance of Fungal Proteins

The literature on consumer response to mycelium from edible mushroom fungi is scarce. In part, this may be caused by the lingering assumption since the 1980s that consumers will not accept such products,^30^ even though certain species may offer consumer benefits. The 1980s were a different era of food production as food safety was still an important point of contention in European and other high-income countries. With improved food technology and associated food safety, consumer demand has since shifted toward the demand for more natural and sustainable food products.

There are some food products based on fungal mycelium on the market. These are not based on mushroom-forming fungi (basidiomycetes) but contain mycelium from ascomycetes. Quorn (mycelium of the fungus *F. venenatum*) was launched in the early 1980s. Surprisingly little attention was paid to acceptance of mycelium as the origin of Quorn. Instead, the focus has been on its marketing as a healthy and tasty meat alternative. At that time, the market for meat alternatives was limited, but nowadays it shows more variation.

There is evidence that molds and fungi associated with foods contribute to the feeling of disgust due to their association to food spoilage, strengthening the assumption that mycelium products could be disgusting to consumers.^30^ To date there is scarce evidence that this is truly the case for fungi grown in controlled situations. In fact, in a study on a fungi patty grown from stale bread it was found that participants did not exhibit disgust.^31^ However, in this study the setup was clearly experimental and relied on volunteers and can hence not be generalized to the general population. The stale bread study does, however, raise the point of substrate. One of the sustainability claims for growing mycelia is that it would be able to repurpose often hard to use side streams at relatively high value. Yet to our knowledge no consumer research on the effect of distinct substrates for mycelia is reported. Even the common practice of growing the common Button mushroom (*A. bisporus*) on composted horse dung has attracted almost no attention, although a disgust response is to be expected.^32^ Disgust is arguably lower for mushrooms, that can be harvested outside the substrate, which cannot be extended to mycelia. For mycelium it is harder, if not impossible, to separate the fungus from its substrate compared to harvesting the mushrooms and consumer studies are needed to investigate whether people have issues with this.

Processing mycelium to remove its substrate may not fully counter disgust response. In addition, processing mycelium protein may result in different consumer issues. Evidence on processing fungal-based material, for example Oyster mushroom stems, suggests that consumers are tolerant to the use of powdered mushroom products.^33^ Currently the focus lies on clean labels, minimally processed products, and ingredient lists. On the other hand, there is a demand for new processing technologies that are developed in a societally acceptable way.^34^ Examples are non-chemical processing technologies such as PEF that may be used to open cells to extract proteins from mycelium. These methods are considered natural by consumers, albeit perceived with some general level of techno-scepticism.^35^ Whether and in what application consumer opinion favors unprocessed mycelia with the substrate still attached, or purified, but processed, mycelium protein remains to be seen.

Besides these mycelium specific issues, distrust in the food industry and scepticism toward claims made in relation to health and sustainability result from the growing abstraction of food ingredients (e.g., E-numbers) and the distance between food production practices and consumers. Distrust may result from the reluctance of food industry and governments to stand up for consumer concerns against vested interests of industry (e.g., acrylamide, artificial colorants). Given the controversial protein assessments for mycelium, some companies may overpromise on the products.^36^ For example, to describe Quorn, the term mycoprotein is often used. This would suggest that the product is mainly protein; however, it includes all components of the mycelium, including carbohydrates and chitin, next to proteins. Such actions may also invoke distrust and scepticism that will harm not only the proposed products but also a nascent mycelium food sector as a whole. Whether this will matter in this specific case remains unclear and may depend on many factors, including choice of substrate, level and type of processing, and claims made.

Another issue that needs to be considered when developing food alternatives is the question of what consumers ultimately want. Consumers buy food products for the experience they have when preparing and consuming them. These experiences are often related to ease of use and preparation, fit within existing patterns, alignments with ideals, convenience of purchase and storing, and, of course, price, taste, and texture, often more so than with functional properties.^37^

## 6 Concluding Remarks and Prospects

This Perspective outlines the most important available knowledge and gap needed to produce food grade protein from lignocellulosic side streams with mycelium of basidiomycetes. A first observation is that the potential of using mycelium of mushroom forming fungi cultured on lignocellulose appears to be substantial. Currently only about 80 mushroom species are or can be cultivated at a commercial level. If the protein production is not directed at the fruiting bodies but to the mycelium that is far more feasible to grow, a tremendous number of species remain to be explored. Excluding ectomycorrhizal species (∼6000) that are often difficult to grow in the absence of their host and assuming that 20% of all mushroom species will turn out to be inedible or poisonous, more than 11,000 candidate mushroom species would remain (based on 20,000 mushroom species). Clearly most mushroom species that could serve as a source of protein remain thus to be examined. Some of these may be more efficient and contain more protein than the ones currently used for consumption. In addition, studying the effect of different substrates and additives on protein production in various species may result in improved species/substrate combinations.

The use of lignocellulosic rich side streams is a promising way to grow mycelia as that would provide a high-level reuse of side streams that are not easily up-valued otherwise. While this claim sounds appealing, current practice from mushroom cultivation on lignocellulosic rich streams involves slow growing species which reduces economic viability of such systems or depends on substrates supplemented with other (food grade) nutrients. If these additional nutrients are produced for the purpose of enriching mushroom substrate, they are likely to reduce sustainability gain. Nevertheless, we consider it a relevant venue for future research to investigate whether mixing different side streams (e.g., ammonia from animal husbandry) with woody side streams could achieve good growth speeds or higher protein levels, combined with full usage of lignocellulose.

In this context, food safety issues need to be considered. It should be determined if and which harmful components are present in the substrates, such as theobromine in cocoa husks or heavy metals. This is also dependent on whether these components end up in the mycelium or in the protein extracts and whether they are easily removed during purification. Also of importance in this respect are metabolites/compounds made by the fungus that may either be healthy or harmful. While edible mushrooms are generally regarded as safe on approved substrates, it is unclear whether this extends to their mycelia and to food safety of mushroom and mycelia grown on novel side streams.

The issue of substrate choice becomes even more relevant when assessing harvesting and processing of mycelia. Current mycelium products circumvent this issue by using food grade substrates (liquid fermentation mainly) or by shaving off mycelium growing on the outside of the substrate. To unlock the full potential of mycelium, it would be worthwhile to also harvest mycelium grown in the substrate via solid state fermentation. This raises several questions that need to be further studied: (1) do we need to separate proteins and amino acids from the substrate, (2) if needed, which processing steps can we use to separate the proteins and amino acids from the substrate, and (3) to what extent can non-food-grade substrate be converted to edible food grade ingredients that are not only edible physiologically but also accepted by the market and regulation.

For the determination of the amount of protein produced, the current methods limit a good comparison between different studies. This hampers relevant assessment of real protein, amino acids, and nutritional values of mycelium. We suggest determining the total and free AA content together with specified cultivation conditions. This way, the effect of the different types of substrates for growing mycelium and the production of protein can be compared. The type and yield of proteins present in/produced by mycelia will determine its application such as nutritional source or for its technofunctional properties in food.

The economic viability of producing food grade proteins from lignocellulosic side streams by mycelium from basidiomycetes depends on different factors such as the amount of protein produced and harvested. The species of basidiomycete in combination with the lignocellulosic side stream, possibly supplemented with a nitrogen source that may increase protein quality and yield, will be of high importance. Furthermore, it is important to know if the remaining side stream after fermentation can be valorized. The effect of these different factors should be answered in more detail to gain more insight into the economic viability of the process. If proteins are extracted, the remaining materials must be valorized for its total use. For example, chitin can be extracted as a vegan source for chitosan.

The consumer angle to marketing mycelium products is also one with many open questions. How will consumers appreciate mycelia, either unprocessed or as an ingredient. How should the products be positioned within the larger assortments in retail, what do consumers demand of such products, and how will associations with molds and fungi, unnaturalness due to heavy processing, the choice of substrates, and scepticism of the food industry influence the introduction of such products? All these open questions require further study to make the best possible entry into the market for mycelium products.

We conclude that there is huge potential for proteins from mycelium grown on lignocellulosic side streams. This requires a major shift in thinking about both fungal cultivation and the use of lignocellulosic side streams for food which will require substantial additional research and reconsideration of current practices. It is worthwhile to set forth on such a program to unlock the potential of proteins produced by mycelia from basidiomycetes on otherwise underutilized but abundant side streams.
